# Supplementary figures and images for: Cimicifuga racemosa Extract Ze 450 Re-Balances Energy Metabolism and Promotes Longevity
Source: Antioxidants (Basel). 2021 Sep 8;10(9):1432. doi: 10.3390/antiox10091432 (PMC8466145; doi:10.3390/antiox10091432)

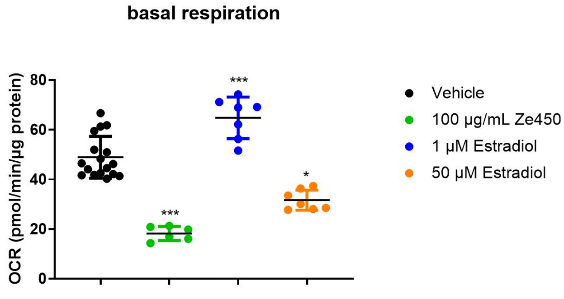

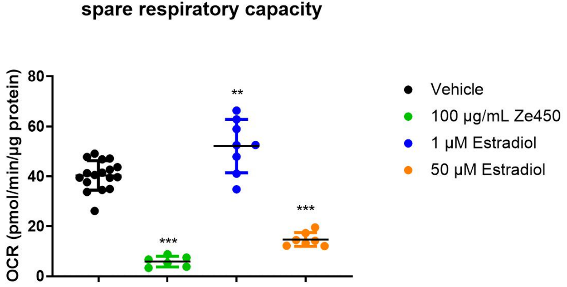

Supplement: Supplementary file 1 [file antioxidants-10-01432-s001.zip › antioxidants-1326127-supplementary/Figure S5.docx]
